# Null Distance and Convergence of Lorentzian Length Spaces

**DOI:** 10.1007/s00023-022-01198-6

**Published:** 2022-05-31

**Authors:** Michael Kunzinger, Roland Steinbauer

**Affiliations:** Faculty of Mathematics, University of Vienna, Oskar-Morgenstern-Platz 1, 1090 Wien, Austria

**Keywords:** 53C23, 53C50, 53B30, 51K10, 53C80

## Abstract

The null distance of Sormani and Vega encodes the manifold topology as well as the causality structure of a (smooth) spacetime. We extend this concept to Lorentzian length spaces, the analog of (metric) length spaces, which generalize Lorentzian causality theory beyond the manifold level. We then study Gromov–Hausdorff convergence based on the null distance in warped product Lorentzian length spaces and prove first results on its compatibility with synthetic curvature bounds.

## Introduction

Metric geometry [6, 7] has led to identifying the ‘metric core’ of many results in Riemannian differential geometry, to clarifying the interdependence of various concepts, and to generalizations of central notions to lower regularity. In particular, Riemannian manifolds carry a natural metric structure and standard notions of convergence such as Gromov–Hausdorff (GH) and Sormani–Wenger intrinsic flat (SWIF) convergence [19] interact well with geometric quantities, in particular with curvature bounds.

Despite the increasing demand for a Lorentzian analog of this framework, particularly driven by general relativity (GR), see, e.g., [9], a comparable metric theory is still in its infancy. Only recently Sormani and Vega [18] put forward a solution to one prime obstacle, i.e., the fact that the Lorentzian distance does not induce a metric structure.^1^ They constructed a ‘null distance’ capable of encoding both the topological and the causality structure of the manifold, and first convergence results built on the corresponding metric and integral current structures were established by Burtscher and Allen [4].

In a somewhat parallel approach more directly rooted in GR, Kunzinger and Sämann [14] introduced a notion of Lorentzian length spaces. Based on the time separation function, this construction provides a close Lorentzian analog of (metric) length spaces. In particular, it allows one to extend beyond the manifold level the synthetic approach to (sectional) curvature bounds, which was introduced for general semi-Riemannian manifolds by Alexander and Bishop [2].

In this work, we provide a natural next step toward a comprehensive notion of metric limits for Lorentzian manifolds by considering GH convergence based on the null distance in the class of Lorentzian length spaces, thereby extending the results in [4] beyond the manifold level. Furthermore, we show that in certain warped product Lorentzian length spaces GH convergence interacts well with synthetic curvature bounds.

This work is structured in the following way: We collect preliminaries on the null distance, the convergence results of [4], Lorentzian synthetic curvature bounds, and Lorentzian length spaces in Sect. 2. Then, in Sect. 3, we extend the null distance to the setting of Lorentzian (pre-)length spaces and, following [4, 18], establish its fundamental properties. Finally, in Sect. 4, we study GH limits of warped product Lorentzian length spaces and prove or main results on their interaction with curvature bounds.

In the remainder of this introduction, we collect some basic notions and conventions. All manifolds are assumed to be smooth, connected, Hausdorff, second countable, of arbitrary dimension $n\ge 2$, and without boundary. A spacetime (*M*, *g*) is a time-oriented Lorentzian manifold, where we use the signature $(-,+,\dots ,+)$. We will deal with metrics of various regularity, but generally assume them to be smooth unless explicitly stated otherwise. Causality notions will be based on locally Lipschitz curves, and we denote the *chronological* and the *causal* relation by *I* and *J* and write $p\ll q$ and $p\le q$ if $q\in I^+(p)$ and $p\in J^+(q)$, respectively. A *generalized time function* $\tau :M\rightarrow \mathbb {R}$ is a function that is strictly increasing along all future-directed causal curves. It is called a *time function* if it is continuous. For points $p,q\in M$, the *time separation function* $\rho (p,q)$ is the supremum of the length of all future-directed causal curve segments from *p* to *q* with the understanding that $\rho (p,q)=0$ if there is no such curve, i.e., if $q\not \in J^+(p)$. In all matters of semi-Riemannian geometry and causality theory, we will adopt the conventions and notations of [17] and [16].

Finally, we will often deal with *warped products* $M=B\times _f F$, where $f>0$ is the warping function and $(B,g_B)$ and $(F,g_F)$ are semi-Riemannian manifolds. The metric *g* on $M=B\times F$ is then given by $g=g_B+f^2g_F$, where, as usual, we notationally suppress the projections. We will most of the time deal with the case that the base *B* is a real interval *I* and the fiber $(F,g_F)$ is Riemannian. Then, the warped product metric takes the form $g=-dt^2+f^2g_F$.

## Preliminaries

The null distance of Sormani and Vega [18] provides a way of encoding the manifold topology as well as the causal structure of a spacetime. Given a generalized time function $\tau$ on (*M*, *g*), the null distance is defined by

1 $$\begin{aligned} {\hat{d}}_\tau (p,q)=\inf \{{\hat{L}}_\tau (\beta ):\ \beta \ \text{ piecewise } \text{ causal } \text{ from } p \text{ to } q\}. \end{aligned}$$

Here, the null length of $\beta :[a,b]\rightarrow M$ is given by ${\hat{L}}_\tau (\beta )=\sum _{i=1}^k|\tau (x_i)-\tau (x_{i-1})|$, where $a=s_0<s_1<\dots <s_k=b$, $x_i=\beta (s_i)$ are the break points. It then holds that ${\hat{d}}_\tau$ is a pseudometric on *M* [18, Prop. 3.8] and it is a (conformally invariant) metric that induces the manifold topology if $\tau$ is continuous and locally anti-Lipschitz, i.e., if any point in *M* has a neighborhood *U* with a distance function $d_U$ such that

2 $$\begin{aligned} \forall \ x\le y\ \in U\ \Rightarrow \ \tau (y)-\tau (x)\ge d_U(x,y). \end{aligned}$$

We say that ${\hat{d}}_\tau$ *encodes the causality* of *M* if

3 $$\begin{aligned} x\le y \Leftrightarrow {\hat{d}}_\tau (x,y)=\tau (y)-\tau (x), \end{aligned}$$

a property that is stronger than definiteness [18, Lem. 3.12]. For warped product spacetimes $I\times _f S$, it holds by [18, Thm. 3.25] that the null distance induced by any smooth time function (depending only on $t\in I$) is definite and encodes the manifold topology as well as the causality. Note that the completeness assumption on the fiber is not needed, a fact which also follows from our Theorem 4.11.

The metric and integral current structure of Lorentzian manifolds based on the null distance was further studied in [4]. There, Allen and Burtscher showed that for any spacetime (*M*, *g*) with locally anti-Lipschitz time function $\tau$, $(M,{\hat{d}}_\tau )$ is a length space [4, Thm. 1.1]. They also proved first GH and SWIF convergence results for warped product spacetimes: Given a (connected, compact) Riemannian manifold $(\Sigma ,h)$, they consider sequences of warped product spacetimes $(M=I\times \Sigma ,g_j=-dt^2+f_j^2(t)h)$ where *I* is a closed interval. If the (continuous) warping functions $f_j:I\rightarrow (0,\infty )$ are uniformly bounded away from 0 and if they converge uniformly to a limit function *f*, then the corresponding null distances ${\hat{d}}_j$ converge uniformly to ${\hat{d}}_f$ on $M=I\times \Sigma$ and the metric spaces $(M,{\hat{d}}_{g_j})$ converge to $(M,{\hat{d}}_{g}$) in the GH as well as in the SWIF sense [4, Thm. 1.4]. Here, *g* is the limiting warped product metric $g=-dt^2+f(t)^2h$.

The Lorentzian length spaces of [14] generalize the notion of length space to the Lorentzian world. Let (*X*, *d*) be a metric space and assume *X* is endowed with a preorder $\le$ as well as a transitive relation $\ll$ contained in $\le$, which we call the timelike and causal relation, respectively. If, in addition, we have a lower semicontinuous map^2^$\rho :X\times X \rightarrow [0, \infty ]$ that satisfies the reverse triangle inequality and $\rho (x,y)>0 \Leftrightarrow x\ll y$, then $(X,d,\ll ,\le ,\rho )$ is called a *Lorentzian pre-length space* with *time separation function* $\rho$.

A locally Lipschitz curve on an arbitrary interval $\gamma :I\rightarrow X$ that is non-constant on any sub-interval is called (future-directed) *causal (timelike)* if for all $t_1<t_2\in I$ we have $\gamma (t_1)\le \gamma (t_2)$ ($\gamma (t_1)\ll \gamma (t_2)$). A causal $\gamma$ is called *null* if no two points on the curve are timelike related. The length of a future-directed causal $\gamma :[a,b]\rightarrow X$ is defined via $\rho$ by

$$\begin{aligned} L_\rho (\gamma ):= \inf \left\{ \sum _{i=0}^{N-1} \rho (\gamma (t_i),\gamma (t_{i+1})): a=t_0<t_1<\ldots <t_N=b,\ N\in \mathbb {N}\right\} . \ \end{aligned}$$

We call a future-directed causal curve $\gamma :[a,b]\rightarrow X$ *maximal* if it realizes the time separation, $L_\rho (\gamma ) = \rho (\gamma (a),\gamma (b))$. In analogy with metric length spaces, we call *X* a *Lorentzian length space* if, in addition to some technical assumptions (cf. [14, Def. 3.22]) $\rho = {\mathcal {T}}$, where for any $x,y\in X$ we set

$$\begin{aligned} {\mathcal {T}}(x,y):= \sup \{L_\rho (\gamma ):\gamma \text { future-directed causal from }x \text { to } y\}\,, \end{aligned}$$

if there is a future-directed causal curve from *x* to *y*. Otherwise, we set ${\mathcal {T}}(x,y):=0$.

Causality theory in Lorentzian length spaces [1, 11, 14] extends standard causality theory [16] beyond the spacetime setting, to which it reduces for smooth strongly causal spacetimes. Hence, any smooth strongly causal spacetime is an example of a Lorentzian length space, but more generally, spacetimes with low regularity metrics and certain Lorentz–Finsler spaces [15] provide further examples [14, Sec. 5]. In particular, any continuous spacetime with strongly causal and causally plain metric (a condition that rules out causal pathologies, see [10, Def. 1.16]) is a (strongly localizable, for a definition see below) Lorentzian length space.

Based on pioneering work by Harris [12], Alexander and Bishop in [2] gave a characterization of sectional curvature bounds in terms of triangle comparison in smooth semi-Riemannian manifolds. We say that (*M*, *g*) has *sectional curvature bounded below* by some constant *K*, $R\ge K$, if the sectional curvatures for all spacelike planes are bounded below by *K* and if for all timelike planes they are bounded above by *K*. Equivalently, we have

4 $$\begin{aligned} R\ge K\quad \text{ if }\quad R(v,w,v,w)\ge K\left( \langle v,v,\rangle \langle w,w,\rangle -\langle v,w\rangle ^2\right) . \end{aligned}$$

Then, it holds [2, Thm. 1.1] that $R\ge K$ ($R\le K$) if and only if in any convex (totally normal) neighborhood the signed length of the geodesic between two points on a geodesic triangle is at least (at most) that of the corresponding points in the model triangle in ${\mathbb {L}}^2(K)$. Here, the *signed length* of a geodesic in a convex neighborhood is defined as the signed length of the connecting vector $|\gamma _{pq}|_\pm =\text {sign}(\gamma _{pq})\sqrt{|\langle \gamma _{pq},\gamma _{pq}\rangle |}$, with the sign of timelike vectors taken to be negative. Moreover, the Lorentzian model spaces ${\mathbb {L}}^2(K)$ of constant sectional curvature *K* are

5 $$\begin{aligned} {\mathbb {L}}^2(K) = \left\{ \begin{array}{ll} {\tilde{S}}^2_1(r) &{}\quad K=\frac{1}{r^2}\\ \mathbb {R}^2_1 &{}\quad K=0\\ {\tilde{H}}^2_1(r) &{}\quad K= -\frac{1}{r^2}, \end{array} \right. \end{aligned}$$

where ${\tilde{S}}^2_1(r)$ is the simply connected covering manifold of the two-dimensional *Lorentzian pseudosphere* $S^2_1(r)$, $\mathbb {R}^2_1$ is two-dimensional *Minkowski space*, and $\tilde{H}^2_1(r)$ is the simply connected covering manifold of the two-dimensional *Lorentzian pseudohyperbolic* space.

Again, in parallel to the case of metric geometry, appropriate notions of synthetic (timelike or causal) curvature bounds based on triangle comparison have been introduced in Lorentzian length spaces. By a timelike geodesic triangle, we mean a triple $(x,y,z)\in X^3$ with $x\ll y \ll z$ such that $\rho (x,z)<\infty$ and such that the sides are realized by future-directed causal curves. We then say, cf. [14, Def. 4.7], that a Lorentzian pre-length space $(X,d,\ll ,\le ,\rho )$ has *timelike curvature bounded below* (above) by $K\in \mathbb {R}$ if every point in *X* has a so-called comparison neighborhood *U* such that:

(i) $\rho |_{U\times U}$ is finite and continuous.
(ii) Whenever $x,y \in U$ with $x \ll y$, there exists a causal curve $\alpha$ in *U* with $L_\rho (\alpha ) = \rho (x,y)$.
(iii) If (*x*, *y*, *z*) is a timelike geodesic triangle in *U*, realized by maximal causal curves $\alpha , \beta , \gamma$ whose side lengths satisfy the appropriate size restrictions (see [14, Lem. 4.6]), and if $(x',y',z')$ is a comparison triangle of (*x*, *y*, *z*) in ${\mathbb {L}}^2(K)$ realized by timelike geodesics $\alpha '$, $\beta '$, $\gamma '$, then whenever *p*, *q* are points on the sides of (*x*, *y*, *z*) and $p', q'$ are corresponding points^3^ of $(x',y',z')$, we have $\rho (p,q)\le \rho '(p',q')$ (respectively, $\rho (p,q)\ge \rho '(p',q'))$.

We close this section by recalling further central notions in Lorentzian pre-length spaces. Generally, causality conditions such as strong causality and global hyperbolicity are translated in perfect analogy from the spacetime setting. $(X,d,\ll ,\le ,\rho )$ is called *causally path connected* if for all $x,y\in X$ with $x\ll y$ and all *x*, *y* with $x\le y$ there is a future-directed timelike resp. causal curve from *x* to *y*. A *localizing neighborhood* $\Omega _x$ of a point $x\in X$ is a substitute for a convex neighborhood, and a Lorentzian pre-length space is called *localizable* if every *x* has such a neighborhood. It is defined by the conditions:

(i) There is a $C>0$ such that $L^d(\gamma )\le C$ for all causal curves $\gamma$ contained in $\Omega _x$ (we say that *X* is *d*-compatible).
(ii) There is a continuous map $\omega _x:\Omega _x \times \Omega _x\rightarrow [0,\infty )$ such that $(\Omega _x, d|_{\Omega _x\times \Omega _x}, \ll |_{\Omega _x\times \Omega _x}, \le |_{\Omega _x\times \Omega _x}, \omega _x)$ is a Lorentzian pre-length space with the following non-triviality condition: For every $y\in \Omega _x$, we have $I^\pm (y)\cap \Omega _x\ne \emptyset$.
(iii) For all $p,q\in \Omega _x$ with $p<q$, there is a future-directed causal curve $\gamma _{p,q}$ from *p* to *q* that is maximal in $\Omega _x$ and satisfies
  6 $$\begin{aligned} L_\tau (\gamma _{p,q}) = \omega _x(p,q) \le \tau (p,q)\,. \end{aligned}$$

If, in addition, the neighborhoods $\Omega _x$ can be chosen such that

(iv) Whenever $p,q\in \Omega _x$ satisfy $p\ll q$, then $\gamma _{p,q}$ is timelike and strictly longer than any future-directed causal curve in $\Omega _x$ from *p* to *q* that contains a null segment,

then $(X,d,\ll ,\le ,\rho )$ is called *regularly localizable*. Finally, if every point $x\in X$ has a neighborhood basis of open sets $\Omega _x$ satisfying (i)–(iii), respectively, (i)–(iv), then $(X,d,\ll ,\le ,\rho )$ is called *strongly localizable*, respectively, *SR-localizable*.

In a strongly causal and localizable Lorentzian pre-length space, the length $L_\rho$ is upper semicontinuous, if it is regularly localizable, maximal causal curves have a causal character, and the push-up principle holds [14, Prop. 3.17, Thms. 3.18, 3.20]. A Lorentzian pre-length space is called *geodesic* if for all $x<y$ there is a future-directed causal curve $\gamma$ from *x* to *y* with $\tau (x,y)=L_\tau (\gamma )$ (hence maximizing). Any globally hyperbolic Lorentzian pre-length space is geodesic [14, Thm. 3.30].

## The Null Distance in Lorentzian Length Spaces

In this section, we extend the notion of null distance to the setting of Lorentzian (pre-)length spaces and establish its fundamental properties.

### Definition 3.1

Let $(X,d,\ll ,\le ,\rho )$ be a Lorentzian pre-length space. A map $\tau : X\rightarrow \mathbb {R}$ is called a *generalized time function* if $\tau$ is strictly increasing along every (non-trivial) future-directed causal curve. If $\tau$ is continuous, it is called a *time function*.

As we shall see in Theorem 3.13, existence of a ‘reasonable’ time function implies strong causality. While a significant part of the causal ladder for Lorentzian length spaces has been established in [1, 14], the precise relationship between existence of time functions and stable causality in this general framework is still an open question.^4^

### Definition 3.2

A map $\beta : [a,b] \rightarrow X$ from a closed interval into a Lorentzian pre-length space *X* is called a *piecewise causal curve* if there exists a partition $a=s_0<s_1<\dots<s_{k-1}<s_k=b$ such that each $\beta _i:=\beta |_{[s_{i-1},s_i]}$ is either trivial (i.e., constant) or future-directed causal or past-directed causal. Given, in addition, a generalized time function $\tau : X\rightarrow \mathbb {R}$, the *null length* of $\beta$ is

7 $$\begin{aligned} {\hat{L}}_\tau (\beta ) := \sum _{i=1}^k |\tau (x_i)-\tau (x_{i-1})|, \end{aligned}$$

where $x_i=\beta (s_i)$ ($i=0,\dots ,k$). Moreover, for $p, q\in X$ we define the *null distance* of *p* and *q* by

8 $$\begin{aligned} {\hat{d}}_\tau (p,q) := \inf \{{\hat{L}}_\tau (\beta ) \mid \beta \text { piecewise causal from } p \text { to } q \}. \end{aligned}$$

### Remark 3.3

Contrary to the convention used in [18, Def. 3.1], causal curves in Lorentzian pre-length spaces are always assumed to be nowhere constant (in accordance with the common custom in general relativity). To obtain a faithful generalization of null length and null distance from [18] to our setting, we therefore explicitly allowed constant (sub-)curves in the definition of piecewise causal curves above (although excluding them would not change the $\inf$ in 8).

### Definition 3.4

A Lorentzian pre-length space is called *sufficiently causally connected* (scc) if it is path connected, causally path connected and if every point $p\in X$ lies on some timelike curve.

Under this assumption, we indeed have the following fundamental existence result:

### Lemma 3.5

Let $(X,d,\ll ,\le ,\rho )$ be an scc Lorentzian pre-length space. Then, for any $p, q\in X$ there exists a piecewise causal curve from *p* to *q*.

### Proof

By [14, Lemma 2.12], each $I^\pm (x)$ ($x\in X$) is open, and by causal path connectedness, the relation $p\ll q$ is always realized by the existence of a future-directed timelike curve from *p* to *q*. Moreover, since any $p\in X$ lies on a timelike curve, we have $X=\bigcup _{x\in X} I^-(x) \cup \bigcup _{y\in X} I^+(y)$. Based on these observations, a straightforward adaptation of the proof of [18, Lemma 3.5] yields the claim. The only difference is that in the present situation the finite covering of any path from *p* to *q* will in general contain both timelike futures and timelike pasts of points in *X*.^5^$\square$

We also have the following analog of [18, Lemma 3.6]:

### Lemma 3.6

Let $\tau$ be a generalized time function on a Lorentzian pre-length space and let $\beta : [a,b]\rightarrow X$ be piecewise causal from *p* to *q*. Then,

(i) ${\hat{L}}_\tau (\beta ) = 0$ if and only if $\beta$ is trivial.
(ii) ${\hat{L}}_\tau (\beta ) = \tau (q) - \tau (p)$ if and only if $\beta$ is future-directed causal or constant.
(iii) $${\hat{L}}_\tau (\beta ) \ge \max _{y\in \beta } \tau (y) - \min _{x\in \beta }\tau (x) \ge |\tau (q)-\tau (p)|$$.
(iv) If $\tau \circ \beta : [a,b]\rightarrow X$ is absolutely continuous, then
  $$\begin{aligned} {\hat{L}}_\tau (\beta ) = \int _a^b |(\tau \circ \beta )'|(s)\,ds. \end{aligned}$$

### Proof

By [14, Def. 2.18] (cf. Remark 3.3), causal curves are always assumed to be non-constant, so (i) follows. The other properties are direct consequences of the definitions. $\square$

The following result collects basic properties of the null distance (cf. [18, Lemma 3.8]).

### Lemma 3.7

Let $\tau$ be a generalized time function on an scc Lorentzian pre-length space. Then, the null distance ${\hat{d}}_\tau$ is a finite pseudometric.

### Proof

Symmetry and triangle inequality are immediate from the definition, and finiteness follows from Lemma 3.5. That ${\hat{d}}_\tau (p,p)= 0$ is seen by considering the constant (hence piecewise causal) curve $\beta \equiv p$. $\square$

Since the previous proof relied on Lemma 3.5, we will henceforth usually assume the scc property in order to avoid degenerate situations. The next result corresponds to Lemmas 3.10–3.13 and 3.16–3.18 in [18] (with identical proofs):

### Proposition 3.8

Let $\tau$ be a generalized time function on an scc Lorentzian pre-length space *X*. Then,

(i) For any $p, q\in X$, ${\hat{d}}_\tau (p,q)\ge |\tau (q)-\tau (p)|$. In particular, ${\hat{d}}_\tau (p,q)=0$ implies $\tau (p)=\tau (q)$.
(ii) If $p\le q$, then ${\hat{d}}_\tau (p,q) = \tau (q)-\tau (p)$.
(iii) $\tau$ is bounded on causal diamonds: $p\le x\le q \Rightarrow \tau (p)\le \tau (x)\le \tau (q)$.
(iv) ${\hat{d}}_\tau$ is bounded on causal diamonds: $p\le x, y\le q \Rightarrow {\hat{d}}_\tau (x,y)\le 2(\tau (q) -\tau (p))$.
(v) If *X* has the property that $p\le q \Leftrightarrow {\hat{d}}_\tau (p,q) = \tau (q)-\tau (p)$, then ${\hat{d}}_\tau$ is definite.
(vi) If $\tilde{\tau }$ is another generalized time function on *X* and $\lambda \in (0,\infty )$, then ${\hat{d}}_{\tilde{\tau }}= \lambda {\hat{d}}_{\tau } \Leftrightarrow \tilde{\tau }= \lambda \tau + C$ for some constant *C*.

The following is an analog of [18, Prop. 3.14], whose proof, however, needs to be adapted to the current setting.

### Proposition 3.9

Let $\tau$ be a generalized time function on an scc Lorentzian pre-length space *X*. Then, the following are equivalent:

(i) $$\tau : X\rightarrow \mathbb {R}$$ is continuous.
(ii) ${\hat{d}}_\tau : X\times X \rightarrow \mathbb {R}$ is continuous.

### Proof

(i)$\Rightarrow$(ii): Let $p,q\in X$. Due to $|{\hat{d}}_\tau (p,q) - {\hat{d}}_\tau (p',q')| \le {\hat{d}}_\tau (p,p') + {\hat{d}}_\tau (q,q')$, it suffices to show that we can find an open neighborhood of *p* on which ${\hat{d}}_\tau (p,p')$ becomes arbitrarily small—the same reasoning then applies to $q, q'$. Since *X* is ssc, *p* lies on a timelike curve. Contrary to the manifold setting of [18], however, it might not be an interior point of any such curve, which prevents us from arguing with a timelike diamond as in the proof of [18, Prop. 3.14]. However, by symmetry we may without loss of generality assume that there is a future-directed timelike curve $\alpha _p: [-\delta _0,0] \rightarrow X$ with $\alpha _p(0)=p$. Since timelike futures and pasts are open in any Lorentzian pre-length space ([14, Lemma 2.12]) and since we assume $\tau$ to be continuous, it follows that, for any $0<\delta <\delta _0$, the set

$$\begin{aligned} U_\delta := \{p'\in X \mid |\tau (p) - \tau (p')| < \delta \} \cap I^+(\alpha _p(-\delta )) \end{aligned}$$

is an open neighborhood of *p*. Any $p'\in U_\delta$ can be connected via a timelike curve to $\alpha _p(-\delta )$ and hence can, via $\alpha _p$, be connected to *p* by a piecewise causal curve. By definition of ${\hat{d}}_\tau$ and $U_\delta$, we therefore obtain

$$\begin{aligned} {\hat{d}}_\tau (p,p')&\le |\tau (p') - \tau (\alpha _p(-\delta ))| + |\tau (p) - \tau (\alpha _p(-\delta ))| \\&\le \delta + 2|\tau (p) - \tau (\alpha _p(-\delta ))|. \end{aligned}$$

Consequently, by choosing $\delta$ sufficiently small we can make ${\hat{d}}_\tau (p,p')$ as small as desired.

(ii)$\Rightarrow$(i): By time symmetry, this works exactly as in the proof of [18, Prop. 3.14], but we give the argument for the sake of completeness. Fixing $q_0\in X$, by ssc there is either a $p_0\in I^+(q_0)$ or a $p_0\in I^-(q_0)$, and it will suffice to consider the first possibility. Then, by Proposition 3.8 (ii), for any *p* in the open neighborhood $I^-(p_0)$ of $q_0$ we have $\tau (p) = \tau (p_0) - {\hat{d}}_\tau (p_0,p)$, which is continuous by assumption. $\square$

The following result is a direct generalization of [18, Prop. 3.15]. For the reader’s convenience, we adapt its proof to the current, topologically more general, setup.

### Proposition 3.10

Let $(X,d,\ll ,\le ,\rho )$ be an scc Lorentzian pre-length space with generalized time function $\tau$ and suppose that (*X*, *d*) is locally compact. Then, the following are equivalent:

(i) ${\hat{d}}_\tau$ induces the same topology as *d*.
(ii) $\tau$ is continuous and ${\hat{d}}_\tau$ is definite.

### Proof

(i)$\Rightarrow$(ii): By Proposition 3.9, $\tau$ is continuous. Also, since *d* is definite, the topology on *X* is Hausdorff, implying that ${\hat{d}}_\tau$ is definite as well.

(ii)$\Rightarrow$(i): Also in this case, ${\hat{d}}_\tau$ is continuous by Proposition 3.9, and so it only remains to show that the ${\hat{d}}_\tau$-topology ${\mathcal {O}}_\tau$ is finer than the *d*-topology ${\mathcal {O}}_d$. Let $x\in X$ and $\varepsilon >0$ and denote by $B_\varepsilon ^d(x)$ the open $\varepsilon$-ball for *d*. Since *X* is locally compact, we may assume $\varepsilon$ small enough so that $\partial B_\varepsilon ^d(x)$ is compact. We now distinguish two cases: First, if $\partial B_\varepsilon ^d(x) = \emptyset$, then *X* being connected implies $B_\varepsilon ^d(x)=X$, and so, we may pick any $\varepsilon _0>0$ to obtain $B_{\varepsilon _0}^{{\hat{d}}_\tau }(x) \subseteq B_\varepsilon ^d(x)$. Otherwise, $\varepsilon _0:= \min \{{\hat{d}}_\tau (x,y) \mid y\in \partial B_\varepsilon ^d(x) \}$ exists and is strictly positive since ${\hat{d}}_\tau$ is definite. Let $y\not \in B_\varepsilon ^d(x)$ and pick a piecewise causal curve $\beta :[a,b]\rightarrow M$ from *x* to *y*. With *z* the first intersection of $\beta$ with $\partial B_\varepsilon ^d(x)$, let $\beta _0$ be the initial part of $\beta$ from *x* to *z*. Then, ${\hat{L}}_\tau (\beta ) \ge {\hat{L}}_\tau (\beta _0)\ge \varepsilon _0$. Thus, also ${\hat{d}}_\tau (x,y)\ge \varepsilon _0$. We conclude that also in this case $B_{\varepsilon _0}^{{\hat{d}}_\tau }(x) \subseteq B_\varepsilon ^d(x)$, so indeed ${\mathcal {O}}_\tau \supseteq {\mathcal {O}}_d$. $\square$

Our next goal is to establish that the null distance is definite if and only if the generalized time function is anti-Lipschitz. First, for locally compact *X* the following result ([18, Lemma 4.3]) carries over, with identical proof:

### Lemma 3.11

Let $(X,d,\ll ,\le ,\rho )$ be an scc Lorentzian pre-length space with generalized time function $\tau$ such that (*X*, *d*) is locally compact. Let $d_U$ be a definite distance function on the open subset $U\subseteq X$ such that for all $x,y\in U$ we have $x\le y \Rightarrow \tau (y)-\tau (x)\ge d_U(x,y)$. Then, for any $p\in U$ and any $q\in X\setminus \{p\}$, ${\hat{d}}_\tau (p,q)>0$. In particular, ${\hat{d}}_\tau$ is definite on *U*.

Following [18, Def. 4.4], we call a map $f: X\rightarrow \mathbb {R}$ *anti-Lipschitz* on $U\subseteq X$ if there exists a definite distance function $d_U$ on *U* such that for all $x\le y$ in *U* we have $f(y)-f(x)\ge d_U(x,y)$. It is called *locally anti-Lipschitz* if every point in *X* possesses a neighborhood on which *f* is anti-Lipschitz. Such a function then is automatically a generalized time function, and by Lemma 3.11, the corresponding null distance is definite. Together with Proposition 3.8 (ii), we obtain (cf. [18, Prop. 4.5]):

### Proposition 3.12

Let $\tau$ be a generalized time function on a locally compact ssc Lorentzian pre-length space *X*. Then, ${\hat{d}}_\tau$ is definite if and only if $\tau$ is locally anti-Lipschitz.

In the above definition of the anti-Lipschitz property, there is no requirement on the compatibility of the local distance function $d_U$ with the topology on *X* induced by the metric *d*. To introduce such an assumption, we call $f: X\rightarrow \mathbb {R}$ *topologically anti-Lipschitz* on the open set $U\subseteq X$ if there exists a definite distance function $d_U$ on *U* that induces the *d*-topology on *U* and such that for all $x,y\in U$ we have $x\le y \Rightarrow \tau (y)-\tau (x)\ge d_U(x,y)$. The function is called *topologically locally anti-Lipschitz* if every point in *X* is contained in an open set *U* equipped with such a $d_U$. Of course, the simplest (and most relevant) situation is the one where indeed $d_U=d$. As the following result shows, it is the topological anti-Lipschitz property that allows us to position the existence of time functions on the causal ladder of Lorentzian pre-length spaces.

### Theorem 3.13

Let $(X,d,\ll ,\le ,\rho )$ be an scc Lorentzian pre-length space that is equipped with a topologically locally anti-Lipschitz time function $\tau$. Then, for each point $p\in X$ and each neighborhood *V* of *p* there exists a neighborhood $U\subseteq V$ of *p* that is causally convex: If $\gamma : [a,b]\rightarrow U$ is causal and $\gamma (a), \gamma (b)\in U$, then $\gamma ([a,b])\subseteq U$. In particular, if *X* is a Lorentzian length space, then *X* is strongly causal.

### Proof

Fixing $p\in X$ and a neighborhood *V* of *p*, pick *U* and $d_U$ as in the assumption. Denote the closed (resp. open) *d*-ball $\{q \mid d(p,q)\le \delta \}$ (resp. $\{q \mid d(p,q) < \delta \}$) of radius $\delta$ by $D^d_\delta (p)$ (resp. $B^d_\delta (p)$), and analogously for $d_U$ and pick $\delta >0$ such that $D^d_{2\delta }(p)\subseteq U$. Next, let $\varepsilon >0$ be such that $D^{d_U}_{2\varepsilon }(p)\subseteq B^d_\delta (p)$ and define $\varphi _\varepsilon : X\rightarrow \mathbb {R}$ by

$$\begin{aligned} \varphi _\varepsilon (q) := \left\{ \begin{array}{ll} \varepsilon - \frac{1}{2} d_U(p,q) &{}\quad q\in D^{d_U}_{2\varepsilon }(p)\\ 0 &{}\quad \text {otherwise.} \end{array} \right. \end{aligned}$$

Then, $\varphi _\varepsilon$ is continuous, and we claim that $\tau _\varepsilon := \tau + \varphi _\varepsilon$ and $\tau _{-\varepsilon }:= \tau - \varphi _\varepsilon$ are time functions on *X*. Let us verify this for $\tau _\varepsilon$, the proof for $\tau _{-\varepsilon }$ being analogous. If $q_1, q_2\in U$ with $q_1 > q_2$, then noting that $|\varphi _\varepsilon (q_1)-\varphi _\varepsilon (q_2)|\le \frac{1}{2} d_U(q_1,q_2)< d_U(q_1,q_2)$ due to the reverse triangle inequality for $d_U$, we get

$$\begin{aligned} \tau _\varepsilon (q_1) = \tau (q_1) + \varphi _\varepsilon (q_1) \ge \tau (q_2) + d_U(q_1,q_2) + \varphi _\varepsilon (q_1) > \tau _\varepsilon (q_2). \end{aligned}$$

Now, suppose that $q_1> q_2$ with $q_1\not \in U$ and $q_2\in U$. Then, $\tau _\varepsilon (q_1)=\tau (q_1)$ and either $\tau _\varepsilon (q_2)=\tau (q_2)$, in which case we are done since $\tau$ is a time function, or $q_2\in D^{d_U}_{2\varepsilon }(p)$. In the latter case, let $\gamma$ be a future-directed causal curve from $q_2$ to $q_1$ and let ${\bar{q}}$ be an intersection of $\gamma$ with the boundary of $D^{d_U}_{2\varepsilon }(p)$. Then,

$$\begin{aligned} \tau _\varepsilon (q_1) = \tau (q_1) \ge \tau ({\bar{q}}) = \tau _\varepsilon ({\bar{q}}) > \tau _\varepsilon (q_2). \end{aligned}$$

The case $q_1\in U$, $q_2\not \in U$ follows symmetrically.

Now, set $U_\varepsilon (p) := \{q\in X \mid \tau _{-\varepsilon }(q)< \tau (p) < \tau _\varepsilon (q) \}$. Then, $U_\varepsilon (p)$ is an open neighborhood of *p* that is contained in *U*, and we are going to show that $U_\varepsilon (p)$ is causally convex. To this end, first note that

9 $$\begin{aligned} \partial U_\varepsilon (p) \subseteq \{q \mid \tau _\varepsilon (q) = \tau (p) \} \cup \{q \mid \tau _{-\varepsilon }(q) = \tau (p) \} =: \Gamma _\varepsilon \cup \Gamma _{-\varepsilon }. \end{aligned}$$

If $\gamma$ is a future-directed causal curve that intersects $\Gamma _\varepsilon$ at $\gamma (t_0)$, then since $\tau _\varepsilon$ is a time function it follows that $\tau _\varepsilon (\gamma (t))\le \tau _\varepsilon (\gamma (t_0))=\tau (p)$ for $t\le t_0$, so in particular $\gamma (t)\not \in U_\varepsilon$ for $t\le t_0$. Analogously, if $\gamma$ intersects $\Gamma _{-\varepsilon }$ at $\gamma (t_0)$, then $\gamma (t)\not \in U_\varepsilon$ for $t\ge t_0$. Together with (9), this implies that $\gamma$ can enter $U_\varepsilon (p)$ only by passing through $\Gamma _\varepsilon$ and can leave it only through $\Gamma _{-\varepsilon }$. Suppose now that $\gamma : [a,b] \rightarrow X$ is future-directed causal with $\gamma (a), \gamma (b) \in U_\varepsilon (p)$. If $\gamma$ were not entirely contained in $U_\varepsilon (p)$, then by what was just shown there would exist $s<t$ in (*a*, *b*) such that $\tau _{-\varepsilon }(\gamma (s)) = \tau (p) =\tau _\varepsilon (\gamma (t))$. But the then $\tau _\varepsilon (\gamma (s))\ge \tau _{-\varepsilon }(\gamma (s)) = \tau _\varepsilon (\gamma (t))$, contradicting the fact that $\tau$ is a time function.

The final claim follows from [14, Th. 3.26 (iv)]. $\square$

### Example 3.14

Let (*M*, *g*) be a smooth manifold with a continuous, causally plain Lorentzian metric *g* and an arbitrary background Riemannian metric *h*. Alternatively, we may consider a locally Lipschitz proper Lorentz–Finsler space $(M,{\mathcal {F}})$ in the sense of [15] such that for the corresponding cone structure *C* we have ${\mathcal {F}}(\partial C)=0$. In both cases, we then obtain a Lorentzian pre-length space $(M,d^h,\ll ,\le ,\rho )$ (see [14, Sec. 5]). Assume that $\tau$ is an *h*-steep time function on *M* (see [15, Sec. 2.2]). Then, if $\gamma :[a,b]\rightarrow M$ is a future-directed causal curve from *p* to *q*, we have

$$\begin{aligned} \tau (q)-\tau (p) = \int _a^b \mathrm{d}\tau (\dot{\gamma })(t)\,\mathrm{d}t \ge \int _a^b \Vert \dot{\gamma }(t)\Vert _h\,\mathrm{d}t \ge d_h(p,q). \end{aligned}$$

Thus, $\tau$ is topologically locally anti-Lipschitz, and Theorem 3.13 implies that any point in *M* has a neighborhood base consisting of causally convex sets. In both cases, this implies that $(M,d^h,\ll ,\le ,\rho )$ is a Lorentzian length space (cf. [14, Th. 5.12, 5.16]). Of course, for smooth Lorentzian manifolds and even for the much more general Lorentz–Finsler setting this is well known. Indeed, for closed cone structures the existence of a time function implies stable causality, which in turn implies strong causality ([15, Th. 2.30]). It is not known whether an analogous result also holds for Lorentzian length spaces (cf. [1] for the definition of stable causality of Lorentzian length spaces).

### Definition 3.15

A piecewise causal curve $\beta : [a,b]\rightarrow X$ in an scc Lorentzian pre-length space *X* with generalized time function $\tau$ is called *minimal* if it minimizes the null distance, i.e., if ${\hat{d}}_\tau (\beta (a),\beta (b))={\hat{L}}_\tau (\beta )$.

As in [18, Cor. 3.19] it follows directly from Lemma 3.6 (ii) that any causal curve $\beta$ from *p* to $q\ge p$ is minimal with

10 $$\begin{aligned} {\hat{d}}_\tau (p,q) = {\hat{L}}_\tau (\beta ) = \tau (q) - \tau (p). \end{aligned}$$

In other words, causal curves are null distance realizers.

Next, we transfer [18, Lemma 3.20] to the Lorentzian length space setting:

### Proposition 3.16

Let $\beta : [a,b]\rightarrow X$ be a minimal piecewise causal curve in an scc Lorentzian pre-length space *X* with generalized time function $\tau$. Then, either $\beta$ is causal or it is piecewise null, changing direction at each break point. If *X* is, in addition, localizable, then $\beta$ is a piecewise null geodesic.

### Proof

The proof of [18, Lemma 3.20] relying only on the properties that $I^\pm (p)$ is open for any $p\in X$ and that $\tau$ is strictly increasing along future-directed causal curves, shows that $\beta$ has the following property: If $a<s_0<s_1<\dots < s_k =b$ denotes the breaks of $\beta$, then no two points of $\beta |_{[s_{i},s_{i+1}]}$ are timelike related, giving the first claim (cf. [14, Def. 2.18]). Suppose now that *X* is localizable, let $t_0\in (s_i,s_{i+1})$, $x_0:=\beta (t_0)$ and let $\Omega _{x_0}$ be a localizing neighborhood of $x_0$. Pick $\varepsilon >0$ such that $\beta ([t_0-\varepsilon ,t_0+\varepsilon ])\subseteq \Omega _{x_0}$. If $\beta |_{[t_0-\varepsilon ,t_0+\varepsilon ]}$ were not maximizing for $\rho$, then there would exist a causal curve $\gamma _{p,q}$ from $p=\beta (t_0-\varepsilon )$ to $q=\beta (t_0+\varepsilon )$ in $\Omega _{x_0}$ such that $L_\rho (\beta |_{[t_0-\varepsilon ,t_0+\varepsilon ]}) < L_\rho (\gamma _{p,q})$. But then, in particular, $0< L_\rho (\gamma _{p,q})$ and thereby $p\ll q$, a contradiction. Consequently, $\beta |_{[s_{i},s_{i+1}]}$ is a geodesic in the sense of [11, Def. 4.1]. $\square$

Finally, we immediately conclude the analog of [18, Cor. 3.21]:

### Corollary 3.17

Let *X* be an scc Lorentzian pre-length space *X* with generalized time function $\tau$ and suppose that $p, q\in X$ are not causally related. If $\beta$ is a piecewise causal curve from *p* to *q* that contains a timelike subsegment, then there exists a strictly shorter piecewise causal curve $\alpha$ from *p* to *q*, i.e., with ${\hat{L}}_\tau (\alpha ) < {\hat{L}}_\tau (\beta )$.

## Warped Products

Warped products are of fundamental importance in Riemannian and Lorentzian geometry. In the context of general relativity, they are generalizations of Friedmann–Lemaître–Robertson–Walker spacetimes, which serve as basic cosmological models of our universe. Warped products and generalized cones likewise play a fundamental role in length spaces with synthetic curvature bounds. In the context of Lorentzian length spaces, they have been studied in [3], and we refer to this work for further background information. We start with a closer look at the null distance on such spaces.

### Null Distance on Generalized Cones

To begin with, we recall some basics of warped products with one-dimensional basis, the so-called *generalized cones* from [3, Sec. 3,4].

Let (*X*, *d*) be a metric space and $I\subseteq \mathbb {R}$ an interval. Observe that in [3] only the case of open *I* has been investigated. Here, to also allow for the compact case, we keep *I* general and often write $I=\langle a,b\rangle$ if we need to specify the boundary points. Then, set $Y:=I\times X$ and put the product metric on *Y*, i.e., $D((t,x),(t',x')) = |t-t'| + d(x,x')$ for $(t,x),(t',x')\in Y$. Let $f:I\rightarrow (0,\infty )$ be continuous. Let $\gamma :J\rightarrow Y$, $\gamma =(\alpha ,\beta )$, where $\alpha :J\rightarrow I$ and $\beta :J\rightarrow X$ are both absolutely continuous and $\alpha$ is strictly monotonous. Then, the metric derivative $v_\beta$ of the curve $\beta$ exists almost everywhere [5, Thm. 1.1.2] and we call $\gamma$

11 $$\begin{aligned} {\left\{ \begin{array}{ll} \text{ timelike }\\ \text{ null }\\ \text{ causal } \end{array}\right. } \quad \text{ if } \qquad -\dot{\alpha }^2 + (f\circ \alpha )^2 v_{\beta }^2\quad {\left\{ \begin{array}{ll} < 0\\ = 0\\ \le 0\,, \end{array}\right. } \end{aligned}$$

almost everywhere. It is called *future-/past-directed causal* if $\alpha$ is strictly monotonically increasing/decreasing, i.e., $\dot{\alpha }>0$ or $\dot{\alpha }<0$ almost everywhere. The length $L(\gamma )$ of a causal curve $\gamma$ is defined by

$$\begin{aligned} L(\gamma ):= \int _a^b \sqrt{\dot{\alpha }^2 - (f\circ \alpha )^2 v_\beta ^2}\,. \end{aligned}$$

The time separation function $\rho :Y\times Y \rightarrow [0,\infty ]$ (called $\tau$ in [3]) is defined as

12 $$\begin{aligned} \rho (y,y'):=\sup \{L(\gamma ):\gamma \text { future-directed causal curve from } y \text { to } y'\}\,, \end{aligned}$$

if this set is non-empty, and $\rho (y,y'):= 0$ otherwise. The causal and timelike relations $p\le q$ resp. $p\ll q$ are defined as usual via the existence of a causal resp. timelike future-directed curve from *p* to *q*. When $I\times X$ is equipped with this time separation function, we write $Y\equiv I\times _fX$ and call it a *generalized cone* (or warped product with one-dimensional basis) with *warping function* *f*.

According to [3, Prop. 3.26], if $Y=I\times _f X$ is a generalized cone, where (*X*, *d*) is a length space, then $(Y,D,\ll ,\le ,\rho )$ is a Lorentzian pre-length space. By [3, Th. 4.8, Cor. 4.9], we have:

#### Theorem 4.1

Any generalized cone $I\times _f X$, where *I* is open and (*X*, *d*) is a locally compact length space, is a strongly causal Lorentzian length space. If *X* is, in addition, geodesic, then $I\times _f X$ is a regular^6^ Lorentzian length space.

Furthermore, [3, Cor. 4.11, 4.12] gives:

#### Theorem 4.2

Let $I\times _f X$ be a generalized cone such that *I* is open and *X* is either a geodesic length space that is proper or a locally compact, complete length space. Then, $I\times _f X$ is globally hyperbolic.

Generalized cones are automatically equipped with the continuous time function $\tau \equiv t: (t,x) \mapsto t$ (see [3, Lemma 4.2]). Also, if *X* is path connected, $Y=I\times _f X$ is scc. For convenience, in what follows when we write that *X* is a length space we will always tacitly assume that *X* possesses only a single *accessibility component* (cf. [7]), i.e., that any two points in *X* can be connected by a path of finite length. In particular, *X* is always supposed to be path connected. Let us denote the null distance on *Y* corresponding to $\tau =t$ by ${\hat{d}}_f$. By Proposition 3.9, ${\hat{d}}_f$ is continuous.

The following result shows that ${\hat{d}}_f$ is a metric (i.e., definite) if *f* is uniformly bounded below by a positive constant:

#### Proposition 4.3

Let (*X*, *d*) be a length space and let $Y=I\times _f X$ be the corresponding generalized cone. Suppose that for some $f_{\min }\in \mathbb {R}_{>0}$ we have $f_{\min } \le f(t)$ for all $t\in I$. Then, for $p=(t_p,x_p)$, $q=(t_q,x_q)$

13 $$\begin{aligned} {\hat{d}}_f(p,q) = |t(p)&-t(q)| = |t_p-t_q|, \qquad \quad \, q \in J^\pm (p) \end{aligned}$$

14 $$\begin{aligned} f_{\min }\cdot d(x_p,x_q)&\le {\hat{d}}_f(p,q) \qquad \quad \, q\not \in J^\pm (p). \end{aligned}$$

Consequently, ${\hat{d}}_f$ is a metric on *Y* and the time function $\tau$ is locally anti-Lipschitz.

#### Proof

We adapt arguments from [4, Lemma 4.9]. If $q \in J^\pm (p)$ and $\beta$ is a causal curve from *p* to *q* then by (10)

$$\begin{aligned} {\hat{d}}_f(p,q) = {\hat{L}}_t(\beta ) = |t(q) - t(p)|. \end{aligned}$$

Now let $q\not \in J^\pm (p)$ and take (according to Lemma 3.5) a piecewise causal curve $\beta :[a,b]\rightarrow Y$ from *p* to *q*. Let $a=t_0< t_1 <\dots t_k=b$ be a subdivision such that each $\beta _i:=\beta |_{[t_{i-1},t_i]}$ is causal. Changing orientation if necessary and using [3, Lemma 3.13] we may assume that $\beta _i(t) = (t,\alpha _i(t))$ with $\alpha _i$ a locally Lipschitz curve in *X*. Since $\beta _i$ is causal, (11) implies that

$$\begin{aligned} v_{\alpha _i}^2(t)\le \frac{1}{f(t)^2} \le \frac{1}{f_{\min }^2}. \end{aligned}$$

Therefore, for $\alpha : J \rightarrow X$ the concatenation of the $\alpha _i$ we obtain that

$$\begin{aligned} d(x_p,x_q)\le L_d(\alpha )= & {} \sum _{i=1}^k L_d(\alpha _i) = \sum _{i=1}^k \int _{t_{i-1}}^{t_i} v_{\alpha _i}(t)\,\mathrm{d}t\\\le & {} \frac{1}{f_{\min }}\sum _{i=1}^k |t_i - t_{i-1}| = \frac{1}{f_{\min }}{\hat{L}}_t(\beta ). \end{aligned}$$

Taking the infimum over all curves $\beta$ we obtain that $d(x_p,x_q)\le \frac{1}{f_{\min }}{\hat{d}}_f(p,q)$.

It is evident from (13, 14) that ${\hat{d}}_f$ is definite. The final claim then follows from Proposition 3.12. $\square$

#### Corollary 4.4

In addition to the assumptions from Proposition 4.3, let *X* be locally compact. Then, ${\hat{d}}_f$ induces the *D*-topology on $Y=I\times _f X$.

#### Proof

This is immediate from the previous result and Proposition 3.10. $\square$

The proof of Proposition 4.3 can be utilized to show definiteness of the null distance on any generalized cone (generalizing [18, Lemma 3.22]):

#### Proposition 4.5

The null distance ${\hat{d}}_f$ on any generalized cone $I\times _f X$ (with *X* a length space) is definite.

#### Proof

Let $p=(t_p,x_p)\ne (t_q,x_q)=q \in I\times _f X$. If $t_p\ne t_q$, then ${\hat{d}}_f(p,q)\ge |t_p-t_q|>0$ by Proposition 3.8 (i). On the other hand, if $t_p=t_q$ and $x_p\ne x_q$, then there exists some $\delta >0$ such that (at least) a one-sided interval, say $[t_p,t_p+\delta ]$ is contained in *I*. Let $\beta$ be a piecewise causal curve connecting *p* and *q*. If $\beta$ leaves the $\delta$-strip $[t_p,t_p+\delta ]$, then its null length is at least $\delta$. On the other hand, if $\beta$ remains in $[t_p,t_p+\delta ]$, then setting $c:= \min _{t\in [t_p,t_p+\delta ]} f(t) >0$, the proof of Proposition 4.3 shows that ${\hat{L}}_t(\beta ) \ge c d(x_p,x_q) >0$. Taking the infimum over all such $\beta$, we obtain that ${\hat{d}}_f(p,q)\ge \min (c d(x_p,x_q), \delta )>0$ also in this case. $\square$

Let us denote the space of all piecewise causal paths in *Y* by $\hat{{\mathcal {A}}}$. Then, we have:

#### Theorem 4.6

Let (*X*, *d*) be a locally compact length space, $f: I \rightarrow \mathbb {R}^+$ continuous, and let $Y=I\times _f X$ be the corresponding generalized cone. Suppose that *f* is uniformly bounded below by a positive constant. Then $(Y,\hat{{\mathcal {A}}},{\hat{L}}_t)$ defines a length structure on *Y* in the sense of [7, Sec. 2.1.1]. Moreover, ${\hat{d}}_f$ is the intrinsic metric on *Y* with respect to this length structure, i.e., $(Y,{\hat{d}}_f)$ is a length space.

#### Proof

Since ${\hat{d}}_f$ induces the metric topology on *Y* by Corollary 4.4, this follows by an obvious adaptation of the proof of [4, Th. 3.5]. $\square$

#### Lemma 4.7

Let $Y=I\times _f X$ be a generalized cone, where (*X*, *d*) is a length space. Then, for any $p, q\in Y$ there exists a piecewise null curve connecting *p* and *q* (i.e., a piecewise causal curve whose every segment is null).

#### Proof

Let $I=\langle a,b\rangle$ and write $p=(t_p,x_p)$, $q=(t_q,x_q)$, where without loss of generality we assume $t_p\le t_q$. We first consider the case where in fact $t_p = t_q \in (a,b)$ and pick $\delta >0$ such that $[t_p,t_p+\delta ]\subseteq I$. If $x_p=x_q$, there is nothing to do. Otherwise, let $\beta : [0,L] \rightarrow X$ be a unit speed curve connecting $x_p$ to $x_q$. Consider the initial value problem

15 $$\begin{aligned} \left\{ \begin{array}{l} \dot{\alpha }_0(s)\, = f(\alpha _0(s))\\ \alpha _0(0) = t_p. \end{array} \right. \end{aligned}$$

The maximal solution to (15) is given by the inverse of the map $r\mapsto \int _{t_p}^r \frac{1}{f(t)}\,dt$ and is defined on $(a_0,b_0)$, where $a_0 = \int _{t_p}^a \frac{1}{f(t)}\,dt$, $b_0= \int _{t_p}^b \frac{1}{f(t)}\,dt$. Similarly, for $s_1\in [0,L]$ we consider the final value problem

16 $$\begin{aligned} \left\{ \begin{array}{l} \dot{\alpha }_1(s)\, = -f(\alpha _1(s))\\ \alpha _1(s_1) = t_p. \end{array} \right. \end{aligned}$$

The solution $\alpha _1$ of (16) has an analogous explicit description as $\alpha _0$. Combining these formulae for $\alpha _0$ and $\alpha _1$ with the fact that *f* is bounded below and above on $[t_p,t_p+\delta ]$ by positive constants, we conclude that for any $s_1$ sufficiently small the following conditions are satisfied:

- $\alpha _0$ and $\alpha _1$ exist on $[0,s_1]$,
- there exists some ${\bar{s}}\in [0,s_1]$ such that $\alpha _0({\bar{s}}) = \alpha _1({\bar{s}})$, and
- both $\alpha _0$ and $\alpha _1$ take values in $[t_p,t_p+\delta ]$ for all $s\in [0,s_1]$.

Consequently, the broken null curve $\gamma : [0,s_1]\rightarrow Y$, $\gamma (s) = (\alpha (s),\beta (s))$, where

$$\begin{aligned} \alpha (s) = \left\{ \begin{array}{ll} \alpha _0(s) &{}\quad \text {for } s\in [0,{\bar{s}}]\\ \alpha _1(s) &{}\quad \text {for } s\in [{\bar{s}},s_1] \end{array} \right. \end{aligned}$$

connects the point $(t_p,x_p)$ to the point $(t_p,\beta (s_1))$. Starting from this new point, we can iterate the procedure, and by choosing for $s_1$ a suitably small fraction of [0, *L*], we obtain the desired broken null curve connecting *p* and *q* in a finite number of steps.

Suppose now that $t_p<t_q$. By introducing, if necessary, an intermediate point *r* with $t_p< t_r < t_q$ and considering *p*, *r* and *r*, *q* separately, we may assume that at most one of $t_p, t_q$ is a boundary point of *I*, say $t_p$ (and the following argument works just as well if both $t_p$ and $t_q$ lie in the interior of *I*). In this case, the solution to (15) attains all *t*-values between $t_p$ and *b*, hence in particular the value $t_q$, say at $s=s'$. Then, following the null curve $\gamma =(\alpha _0,\beta )$ until $s'$ we obtain a point $(t_q,z)$, which according to the first part of the proof can itself be connected to *q* by a piecewise null curve. $\square$

Based on Lemma 4.7, we can also prove the following dual to Proposition 4.3:

#### Proposition 4.8

Let (*X*, *d*) be a length space and let $Y=I\times _f X$ be the corresponding generalized cone. Suppose that for some $f_{\max }\in \mathbb {R}_+$ we have $f(t)\le f_{\max }$ for all $t\in I$. Then, for $p=(t_p,x_p)$, $q=(t_q,x_q)$

17 $$\begin{aligned} {\hat{d}}_f(p,q)&= |t(p)-t(q)| = |t_p-t_q|, \qquad \quad \, q \in J^\pm (p) \end{aligned}$$

18 $$\begin{aligned} {\hat{d}}_f(p,q)&\le f_{\max }\cdot d(x_p,x_q)\qquad \quad \, q\not \in J^\pm (p). \end{aligned}$$

#### Proof

Equation (17) was already established in Proposition 4.3. To show (18), let $\varepsilon >0$ and choose a unit speed path $\beta : [0,L]\rightarrow X$ connecting $x_p$ to $x_q$ such that $L_d(\beta )=L < d(x_p,x_q)+\varepsilon$. Let us suppose, without loss of generality, that $t_p\le t_q$. As in the proof of Lemma 4.7, we then first construct a null curve $\gamma =(\alpha ,\beta )$ emanating from $x_p$ and reaching an endpoint *r* with $t_r=t_q$. Since $q\not \in J^+(p)$, the explicit description of $J^+(p)$ in [3, Cor. 3.24] shows that $\alpha$ is defined on $[0,L']$ for some $L'<L$, and again as in the proof of Lemma 4.7, we can then extend $\alpha$ to all of [0, *L*] such that $\gamma =(\alpha ,\beta ): [0,L]\rightarrow Y$ is a piecewise null curve connecting *p* and *q*. Denoting the break points of $\gamma$ by $s_0,\dots ,s_k$, we then have

$$\begin{aligned} {\hat{L}}_t(\gamma ) = \sum _{i=1}^k |\alpha (s_i)-\alpha (s_{i-1})|. \end{aligned}$$

Here, since $\gamma$ is null and $v_\beta \equiv 1$, we have $|\alpha (s_i)-\alpha (s_{i-1})| \le \int _{s_{i-1}}^{s_i} |\dot{\alpha }(s)|\,ds \le f_{\max }|s_i-s_{i-1}|$. Consequently,

$$\begin{aligned} {\hat{d}}_f(p,q) \le {\hat{L}}_t(\gamma ) \le f_{\max }\cdot L < f_{\max }(d(x_p,x_q)+\varepsilon ), \end{aligned}$$

giving the claim for $\varepsilon \rightarrow 0$. $\square$

#### Remark 4.9

Combining Propositions 4.3 and 4.8, it follows that if the warping function *f* is bounded from above and below by positive constants, then the null distance ${\hat{d}}_f$ induces on any fiber $\{t_0\}\times X$ of the generalized cone $I\times _f X$ a metric that is (bi-Lipschitz) equivalent to the one induced by the original metric *d* (by $((t_0,x_1),(t_0,x_2))\mapsto d(x_1,x_2)$). In the special case of pure Lorentzian products ($f\equiv 1$), both ${\hat{d}}_1$ and *d* induce the same metric on any fiber. This generalizes [18, Prop. 3.3.] and [4, Lemmas 4.4, 4.9].

We can now use Propositions 4.3 and 4.8 to derive a comparison between the null distance ${\hat{d}}_f$ on $I\times _f X$ and the Lorentzian product $I\times _1 X$. Indeed, we have the following generalization of [4, Prop. 4.10]:

#### Proposition 4.10

Let *I* be an interval, (*X*, *d*) a length space, with corresponding generalized cone $Y=I\times _f X$. Suppose that there exist positive constants $f_{\min }, f_{\max }\in \mathbb {R}_+$ such that $0< f_{\min } \le f(t)\le f_{\max }$ for all $t\in I$. Then, for all $p,q\in Y$ we have

19 $$\begin{aligned} \min (1,f_{\min }) {\hat{d}}_1(p,q) \le {\hat{d}}_f(p,q) \le \max (1,f_{\max }) {\hat{d}}_1(p,q). \end{aligned}$$

#### Proof

Using the above results, this follows by a straightforward adaptation of the proof of [4, Prop. 4.10]. $\square$

#### Theorem 4.11

Let *I* be an interval, (*X*, *d*) a length space, and $f:I\rightarrow \mathbb {R}^+$ continuous. Let $\phi : I\rightarrow J\subseteq \mathbb {R}$ be a strictly monotonically increasing bi-Lipschitz homeomorphism and denote by $\tau$ the time function $\tau (t,x)=\phi (t)$ on $I\times _f X$. Then, for any $p,q \in I\times _f X$ the following are equivalent:

(i) $$p\le q$$.
(ii) $${\hat{d}}_{\tau }(p,q) = \tau (q) - \tau (p)$$.
(iii) $${\hat{d}}_f(p,q) = t(q) - t(p)$$.

If *X* is, in addition, geodesic, then (i)–(iii) are further equivalent to $q\in \overline{I^+(p)}$.

#### Proof

By Proposition 3.8, (i) implies (ii) and (iii). Since (iii) corresponds to the special case $\phi = \mathrm {id}_I$, we are left with showing that (ii) implies (i). To see this, we adapt an argument from [18, Lemma 3.24]. Let $p=(t_p,x_p), q=(t_q,x_q)$ and suppose that ${\hat{d}}_\tau (p,q) = \tau (q) - \tau (p)$. If $t_p=t_q$ then ${\hat{d}}_\tau (p,q)=0$, so $p=q$ and we are done (noting that an easy adaptation of Proposition 4.5 yields that ${\hat{d}}_\tau$ is definite). Thus suppose that $t_p<t_q$. Pick $\varepsilon _0>0$ such that the closed relative $\varepsilon _0$-neighborhood $K_{\varepsilon _0}$ in *J* of the interval $\phi ([t_p,t_q])$ is compact. For any $0<\varepsilon <\varepsilon _0$, let $\beta$ be piecewise causal from *p* to *q* with ${\hat{L}}_\tau (\beta ) \le \tau (q)-\tau (p) +\varepsilon$. From Proposition 3.6 (iii), it follows that $\phi \circ \beta \subseteq K_\varepsilon \subseteq K_{\varepsilon _0}$. Let $\beta =\beta _1\cdots \beta _k$ be a decomposition of $\beta$ into causal bits. By [3, Cor. 3.13] we may parametrize each $\beta _i$ such that $(\pm )\beta _i: [t_i,t_i+\delta _i] \rightarrow I\times X$, $\beta _i(t) = (t,\sigma _i(t))$. Here, $t_1=t_p$, $t_k+\delta _k = t_q$. The concatenation $\sigma$ of the corresponding curves $\sigma _i$ connects $x_p$ to $x_q$, and since $\beta _i$ is causal, for almost every $t\in [t_i,t_i+\delta _i]$ we have $v_{\sigma _i}(t) \le \frac{1}{f(t)}$. Set $c:= \max _{t\in K_{\varepsilon _0}} \frac{(\phi ^{-1})'(t)}{f(\phi ^{-1}(t))}$. We have

$$\begin{aligned} L_d(\sigma )&= \sum _{i=1}^k \int _{t_i}^{t_i+\delta _i} v_{\sigma _i}(t)\,\mathrm{d}t = \sum _{i=1}^k \int _{\phi (t_i)}^{\phi (t_i+\delta _i)} v_{\sigma _i}(\phi ^{-1}(s)) (\phi ^{-1})'(s)\,\mathrm{d}s\\&\le \sum _{i=1}^k \int _{\phi (t_i)}^{\phi (t_i+\delta _i)} \frac{(\phi ^{-1})'(s)}{f((\phi ^{-1})(s))} \,\mathrm{d}s. \end{aligned}$$

Note now that $\sum _{i=1}^k (\phi (t_i+\delta _i) - \phi (t_i)) = \phi (t_q) - \phi (t_q) + ({\hat{L}}_\tau (\beta ) - (\phi (t_q)-\phi (t_p)))$. Decomposing the above integrals in accordance with this identity and recalling the definition of ${\hat{L}}_\tau$, we obtain

$$\begin{aligned} d(x_p,x_q) \le L_d(\sigma )&\le \int _{\phi (t_p)}^{\phi (t_q)} \frac{(\phi ^{-1})'(s)}{f((\phi ^{-1})(s))}\,\mathrm{d}s + c \cdot ({\hat{L}}_\tau (\beta ) - (\phi (t_q)-\phi (t_p)))\\&\le \int _{t_p}^{t_q} \frac{1}{f(t)}\,\mathrm{d}t +c \varepsilon . \end{aligned}$$

Letting $\varepsilon \rightarrow 0$, we conclude that $d(x_p,x_q) \le \int _{t_p}^{t_q} \frac{1}{f(t)}\,dt$, which by [3, Cor. 3.24] means that $p\le q$, giving (i).

For the final claim, we need to show that $\overline{I^+(p)} = J^+(p)$. To this end, note that by [3, Cor. 3.24], *X* geodesic implies that $J^+(p)$ is closed, so $\overline{I^+(p)} \subseteq J^+(p)$. The converse inclusion follows from the explicit description of $I^+(p)$ and $J^+(p)$ in [3, Prop. 3.22] and [3, Cor. 3.24]. $\square$

#### Remark 4.12

(i) In [18, Th. 3.25], the authors consider a Lorentzian warped product manifold $(I\times S,-dt^2+ f^2(t)h)$ with *I* an open interval, $f:I\rightarrow \mathbb {R}^+$ continuous and (*S*, *h*) a complete Riemannian manifold. By constructing a suitable conformal metric, they then show that for any smooth function $\phi :I\rightarrow \mathbb {R}^+$ with $\phi '>0$ the time function $\tau (t,x):=\phi (t)$ still encodes the causality of the warped product. Theorem 4.11 is a generalization of this result to the metric setting.
(ii) The assumptions on $\phi$ in Theorem 4.11 can be slightly relaxed (cf. [3, Lemma 3.6]): It suffices to assume that $\phi$ is absolutely continuous, monotonically increasing and that $\phi ^{-1}$ is absolutely continuous with locally bounded derivative.

### Convergence of Generalized Cones and Curvature Bounds

In [4], convergence of generalized cones based on the null distance has been studied in the spacetime setting. Here, we make use of the results just obtained to extend the respective theory beyond the manifold level. Based on Proposition 4.10, we first derive the following essential result on the convergence of null distances for uniformly converging warping functions, generalizing [4, Prop. 5.1]:

#### Theorem 4.13

Let *I* be an interval, (*X*, *d*) a length space, and $f_j: I \rightarrow \mathbb {R}^+$ ($j\in \mathbb {N}$) a sequence of continuous functions that converge uniformly to some $f: I \rightarrow \mathbb {R}^+$. Suppose that there exists a uniform lower bound *c*, i.e., $0<c \le f_j(t)$ for all $t\in I$ and all $j\in \mathbb {N}$. Then, for the null distances of the corresponding generalized cones $I\times _{f_j} X$, $I\times _f X$ we have: Let $r>0$ and $p_0, q_0 \in I\times X$. Then,

20 $$\begin{aligned} \lim _{j\rightarrow \infty } {\hat{d}}_{f_j}(p,q) = {\hat{d}}_f(p,q) \end{aligned}$$

uniformly on $$B_r^{{\hat{d}}_f}(p_0) \times B_r^{{\hat{d}}_f}(q_0)$$.

#### Proof

This follows by a straightforward adaptation of the proof of [4, Prop. 5.1]. Since homothetic Riemannian metrics induce correspondingly scaled Riemannian distances, when mimicking Step 2 of that proof we can introduce new (constantly rescaled) warping functions ad hoc that produce the required distance functions directly. Due to (11), with these choices also the compatibility of the respective notions of causal curves (implemented in [4, Prop. 5.1] via inclusion relations of causal cones of warped product metrics) is guaranteed. Thus, using (19), all estimates can be carried out unchanged in the present setting.

Moreover, we note that, although in the formulation of [4, Prop. 5.1] only pointwise convergence is asserted, it indeed implies the stronger claim made here. Namely, the arguments laid out there show the following: By uniform convergence, the minimum $f_{\min }$ of *f* on *I* is positive. Given any $\varepsilon \in \big (0,\frac{f_{\min }}{4} \big )$, choose $j_0\in \mathbb {N}$ such that $\Vert f-f_j\Vert _{\infty } < \varepsilon$ for all $j\ge j_0$. Then, for all $p,q\in Y$ and all $j\ge j_0$ we have:

21 $$\begin{aligned} {\hat{d}}_f(p,q) - \varepsilon \Big (1 + \frac{3}{f_{\min }} {\hat{d}}_f(p,q) \Big )\le & {} {\hat{d}}_{f_j}(p,q) \le {\hat{d}}_f(p,q)\nonumber \\&+ \varepsilon \Big (1 + \frac{8\varepsilon }{f_{\min }} + \frac{8}{f_{\min }} {\hat{d}}_f(p,q) \Big ). \end{aligned}$$

Using this, it suffices to observe that the factors of $\varepsilon$ in (21) are uniformly bounded on any ${\hat{d}}_f$-ball of finite radius. $\square$

#### Corollary 4.14

In addition to the assumptions of Theorem 4.13, suppose that *X* is locally compact and that $\mathrm {diam}(I\times _f X,{\hat{d}}_f) < \infty$. Then, the sequence $(I\times _{f_n} X,{\hat{d}}_{f_n})$ of metric spaces converges uniformly to $(I\times _f X,{\hat{d}}_f)$ in the sense of [7, Def. 7.1.5], i.e., ${\hat{d}}_{f_n} \rightrightarrows {\hat{d}}_f$.

#### Proof

Let $F_n =\mathrm {id}: (I\times X,{\hat{d}}_{f_n})\rightarrow (I\times X,{\hat{d}}_f)$. Then, $F_n$ is a homeomorphism since by Corollary 4.4, both ${\hat{d}}_{f_n}$ and ${\hat{d}}_{f}$ induce the same topology as *D* on $I\times X$. Finally, ${\hat{d}}_{f_n} \rightrightarrows {\hat{d}}_f$ by (20) and our assumption on the finite diameter of $(I\times _f X,{\hat{d}}_f)$. $\square$

By Proposition 4.8, the diameter assumption of Corollary 4.14 is satisfied if *X* is a length space, $\mathrm {diam}(X)<\infty$, and either *I* is compact or *I* is bounded and the $f_n$ are additionally uniformly bounded from above.

#### Corollary 4.15

Let *I* be an interval and (*X*, *d*) a length space. Suppose that $f_n:I\rightarrow \mathbb {R}^+$ is a sequence of continuous functions with a positive uniform lower bound that uniformly converges to $f: I\rightarrow \mathbb {R}^+$.

(i) Let *X* be proper and fix $p_0=(t_0,x_0)\in I\times X$. Then $(I\times _{f_n} X,{\hat{d}}_{f_n},p_0) \rightarrow (I \times _f X,{\hat{d}}_f,p_0)$ in the pointed Gromov–Hausdorff sense.
(ii) If both *I* and *X* are compact, then $(I\times _{f_n} X,{\hat{d}}_{f_n}) \rightarrow (I \times _f X,{\hat{d}}_f)$ in the Gromov–Hausdorff sense.

#### Proof

(i) Let $r>0$ and $\varepsilon >0$, then by (20) we can find $N\in \mathbb {N}$ such that for all $n\ge N$ we have (again writing $D^d_r$ for closed *r*-balls with respect to a metric *d*)

22 $$\begin{aligned}&D^{{\hat{d}}_{f_n}}_{(1-\varepsilon )r}(p_0) \subseteq D^{{\hat{d}}_{f}}_{r}(p_0) \subseteq D^{{\hat{d}}_{f_n}}_{(1+\varepsilon )r}(p_0) \end{aligned}$$

23 $$\begin{aligned}&|{\hat{d}}_f(p,q) - {\hat{d}}_{f_n}(p,q)|<\varepsilon \quad \forall p,q\in D^{{\hat{d}}_{f}}_{r}(p_0). \end{aligned}$$

We now define a map $F: D^{{\hat{d}}_{f}}_{r}(p_0) \rightarrow D^{{\hat{d}}_{f_n}}_{r}(p_0)$ as follows: If $p\in D^{{\hat{d}}_{f_n}}_{(1-\varepsilon )r}(p_0)$, we set $F(p):=p$. Otherwise, since $(Y,{\hat{d}}_{f_n})$ is a length space (see Theorem 4.6) and using (22), for any $p\in D^{{\hat{d}}_{f}}_{r}(p_0)$ we can pick some $F(p)\in D^{{\hat{d}}_{f_n}}_{r}(p_0)$ such that ${\hat{d}}_{f_n}(p,F(p))\le \varepsilon$. Then, $F(D^{{\hat{d}}_{f}}_{r}(p_0)) \supseteq D^{{\hat{d}}_{f_n}}_{(1-\varepsilon )r}(p_0)$, hence it is an $\varepsilon$-net in $D^{{\hat{d}}_{f_n}}_{r}(p_0)$. From (23) and the definition of *F*, we conclude that

$$\begin{aligned} |{\hat{d}}_f(p,q) - {\hat{d}}_{f_n}(F(p),F(q))| \le 3\varepsilon \end{aligned}$$

for all $p, q\in D^{{\hat{d}}_{f}}_{r}(p_0)$. Consequently, *F* is a $3\varepsilon$-isometry and thereby

$$\begin{aligned} d_{GH}((D^{{\hat{d}}_{f_n}}_{r}(p_0),p_0),(D^{{\hat{d}}_{f}}_{r}(p_0),p_0))\le 6\varepsilon . \end{aligned}$$

(ii) This follows from (i) and general properties of Gromov–Hausdorff convergence ([13, Prop. 2.4]). Alternatively, we may use Corollary 4.14 and the fact that uniform convergence of compact metric spaces implies Gromov–Hausdorff convergence. $\square$

The following is a compactness result for families of generalized cones:

#### Theorem 4.16

Let *I* be a compact interval and (*X*, *d*) a compact length space. Suppose that ${\mathcal {F}}$ is a family of continuous functions $I\rightarrow \mathbb {R}^+$ that is uniformly bounded above: $\exists C>0$: $f(t)\le C$ for all $t\in I$ and all $f\in {\mathcal {F}}$. Then, ${\mathcal {Y}}:=\{(I\times _f X,{\hat{d}}_f) \mid f\in {\mathcal {F}} \}$ is pre-compact with respect to the Gromov–Hausdorff topology. Thus, any sequence from ${\mathcal {Y}}$ possesses a subsequence that converges in the Gromov–Hausdorff sense.

#### Proof

By compactness of *I* and *X*, for any $\varepsilon >0$ there exists some $N\in \mathbb {N}$ and $\varepsilon$-nets $t_1,\dots ,t_N$ in *I* and $x_1,\dots ,x_N$ in (*X*, *d*). Given any $p = (t_p,x_p)\in I\times X$, there then exist $i,j\in \{1,\dots ,N\}$ such that $|t_p-t_i|<\varepsilon$ and $d(x_p,x_j)<\varepsilon$. By Proposition 4.8, for any $f\in {\mathcal {F}}$ this implies

$$\begin{aligned} {\hat{d}}_f(p,(t_i,x_j)) \le \varepsilon \max (1,C). \end{aligned}$$

Consequently, $\{(t_i,x_j)\mid 1\le i,j \le N\}$ is an $\varepsilon \cdot \max (1,C)$-net for $(I\times _f X,{\hat{d}}_f)$. This shows that ${\mathcal {Y}}$ is uniformly totally bounded (cf. [7, Def. 7.4.13]). The claim then follows from [7, Th. 7.4.15]. $\square$

For the following result, we recall from [3, Ex. 3.31] that the Minkowski cone $\mathrm {Cone}(X)$ over a geodesic length space from [3, Sec. 2] can equivalently be represented as the generalized cone $(0,\infty )\times _{\mathrm {id}} X$. Therefore, the GH-convergence result established below in particular applies to Minkowski cones.

#### Proposition 4.17

Let $(X_n,d_{n},p_n)$ be a sequence of pointed proper length spaces that converge to the pointed proper metric space (*X*, *d*, *p*) in the pointed Gromov–Hausdorff sense, and let *I* be an interval. If all $I\times _{\mathrm {id}} X_n$ have timelike curvature bounded below by 0, then the same is true of $I\times _{\mathrm {id}} X$.

#### Proof

(*X*, *d*) is a length space by [13, Prop. 2.7]. Also, by [3, Th. 2.5], $I\times _{\mathrm {id}} X_n$ has timelike curvature bounded below by 0 if and only if $(X_n,d_n)$ is an Alexandrov space whose curvature is bounded below by $-1$. This metric curvature bound persists through the pointed Gromov–Hausdorff limit of the $(X_n,d_n)$ by [7, Prop. 10.7.1] (and the discussion following [7, Prop. 7.4.12], applied to compact balls containing the quadruples), so appealing again to [3, Th. 2.5] gives the claim. $\square$

#### Remark 4.18

In particular, the conclusion of the Proposition holds if *I*, (*X*, *d*) and all $(X_n,d_n)$ are compact and $(X_n,d_n) \rightarrow (X,d)$ in the Gromov–Hausdorff sense (cf. [13, Cor. 2.5]).

We have the following result on null-distance Gromov–Hausdorff convergence of pure Lorentzian products:

#### Proposition 4.19

Let *I* be a compact interval and let $(X_n,d_n)$ be a sequence of compact length spaces. Let (*X*, *d*) be a compact length space such that $(X_n,d_n) \overset{GH}{\longrightarrow } (X,d)$. Then, $Y_n\equiv (I\times _1 X_n,{\hat{d}}_{n,1}) \overset{GH}{\longrightarrow } (I\times _1 X,{\hat{d}}_{1})\equiv Y$.

#### Proof

We use the characterization of the Gromov–Hausdorff distance via distortions of correspondences (see [7, Sec. 7.3.3]). For any correspondence ${\mathfrak {R}} \subseteq X_n\times X$ between $X_n$ and *X*, we define a correspondence $\hat{{\mathfrak {R}}}\ \subseteq Y_n\times Y$ between $Y_n$ and *Y* by

$$\begin{aligned} \hat{{\mathfrak {R}}} := \{((t,x_n),(t,x)) \in Y_n\times Y \mid t\in I,\ (x_n,x)\in {\mathfrak {R}} \}. \end{aligned}$$

By Propositions 4.3 and 4.8, we have

$$\begin{aligned} {\hat{d}}_1((s,x),(t,y)) = \left\{ \begin{array}{ll} |s-t| &{}\quad (s,x)\in J^\pm (t,y)\\ d(x,y) &{}\quad \text {otherwise.} \end{array} \right. \end{aligned}$$

Here, $(s,x)\in J^\pm (t,y)$ means $d(x,y)\le |s-t|$, so ${\hat{d}}_1((s,x),(t,y)) = \max (d(x,y),|s-t|)$. Analogously, ${\hat{d}}_{n,1}((s,x_n),(t,y_n)) = \max (d_n(x_n,y_n),|s-t|)$. Thus, for the distortions of ${\mathfrak {R}}$ and $\hat{{\mathfrak {R}}}$ we have

$$\begin{aligned}&\mathrm {dis}(\hat{{\mathfrak {R}}}) \\&\quad =\sup \{ |\max (d(x,y),|s-t|)\\&\qquad -\, \max (d_n(x_n,y_n),|s-t|) | \mid ((s,x_n),(s,x)), ((t,y_n),(t,y)) \in \hat{{\mathfrak {R}}}\} \\&\quad \le \sup \{ |d_n(x_n,y_n) - d(x,y)| \mid (x_n,x), (y_n,y) \in {\mathfrak {R}} \} = \mathrm {dis}({{\mathfrak {R}}}). \end{aligned}$$

From this, by [7, Th. 7.3.25] we obtain

$$\begin{aligned} d_{GH}((Y_n,{\hat{d}}_{n,1}),(Y,{\hat{d}}_1))\le & {} \frac{1}{2} \inf _{{\mathfrak {R}}}(\mathrm {dis}(\hat{{\mathfrak {R}}}))\\\le & {} \frac{1}{2} \inf _{{\mathfrak {R}}}(\mathrm {dis}({\mathfrak {R}})) = d_{GH}((X_n,d_n),(X,d)), \end{aligned}$$

giving the claim. $\square$

#### Remark 4.20

Since $D^{{\hat{d}}_1}_r(t,x) = (I\cap [t-r,t+r])\times D^d_r(x)$ (and analogously for closed ${\hat{d}}_n$-balls), an easy modification of the previous proof shows the analogous result for pointed Gromov–Hausdorff convergence: Let *I* be any interval and let $(X_n,d_n,x_n)$ be proper length spaces that converge to the proper metric space (*X*, *d*, *x*) in the pointed Gromov–Hausdorff sense. Then, if $t_n\rightarrow t \in I$, $p_n:=(t_n,x_n)$, $p:=(t,x)$, then also $(I\times _{1}X_n,{\hat{d}}_{n,1},p_n) \rightarrow (I\times _1 X, {\hat{d}}_1,p)$ in the pointed Gromov–Hausdorff sense.

#### Theorem 4.21

Let *I* be a compact interval and let $(X_n,d_n)$ be a sequence of compact length spaces that converge to the compact space (*X*, *d*) in the Gromov–Hausdorff sense. If the timelike curvature of each of the Lorentzian products $I\times _1 X_n$ is non-negative, then the same is true of $I\times _1 X$.

**Remark.** By Proposition 4.19, the assumptions here imply that $(I\times _1 X_n,{\hat{d}}_{n,1}) \overset{GH}{\longrightarrow } (I\times _1 X,{\hat{d}}_{1})$.

#### Proof

Any compact length space is geodesic by the Hopf–Rinow theorem ([6, Prop. I.3.7]). Hence, we may apply [3, Th. 5.7], to conclude from our assumption and the fact that $I\times _1 {{\mathbb {M}}}^2(0)$ has vanishing timelike curvature that the metric curvature of $(X_n,d_n)$ is bounded below by 0. By [7, Prop. 10.7.1], therefore, also the Gromov–Hausdorff limit (*X*, *d*) has non-negative curvature. Since (*X*, *d*) is a length space by [7, Th. 7.5.1] (as well as geodesic by what was said above), [3, Th. 5.7] now yields the claim. $\square$

We also have the following convergence result on (timelike) curvature bounds of generalized cones. For its formulation recall from [2] that a $C^2$-function $f:I\rightarrow (0,\infty )$ is called ($-K'$)-concave (convex), if $f''-K'f\le 0$ ($\ge 0$).

#### Theorem 4.22

Let *I* be compact and assume that $f_n:I\rightarrow (0,\infty )$ is a sequence of ($-K_n'$)-concave functions that converges to $f:I\rightarrow (0,\infty )$ in $C^2$ and such that $K_n'\rightarrow K'$. Let $(X_n,d_n)$ be a sequence of compact length spaces with lower curvature bounds $K_{X_n}\ge K_n:=\sup _{x\in I}(K_n'f_n^2-(f_n')^2)$ converging to the compact space (*X*, *d*) in GH. Then, the generalized cone $Y=I\times _{f} X$ has timelike curvature bounded below by $K'$.

#### Proof

Uniform convergence of $f_n$ and $f_n'$ together with $K_n'\rightarrow K'$ imply that $K_n \rightarrow K:=\sup _{x\in I}(K'f^2-(f')^2)$. Thus, by [7, Thm. 10.7.1], *X*, being the GH-limit of the $X_n$ has curvature bounded below by *K*. Moreover, *f* is ($-K'$)-concave and so by [3, Cor. 5.4] the generalized cone $Y=I\times _f X$ has timelike curvature bounded below by $K'$. $\square$

## Footnotes

1 For a general discussion, see [20].

2 In previous accounts on Lorentzian length spaces, the time separation function was denoted by $\tau$. To comply with our main points of reference [4, 18], we here reserve the letter $\tau$ for time functions.

3 This means that $p'$ lies on the side corresponding to the side containing *p* at the same time separation from the vertex (e.g., if *p* lies on the side *xy*, then $\rho (x,p)=\rho '(x',p')$, etc.). Similarly for $q'$.

4 Note added in proof: In the recent paper [8], the authors have been able to show that under mild additional assumptions the existence of time functions is indeed equivalent to stable causality.

5 Note that this can really occur, e.g., for $[a,b]\times _f X$ in points with $t=a,b$.

6 See [14, Def. 3.22] for a definition.
